# A case report of an adjustable gastric band erosion and migration into the jejunum resulting in biliary obstruction

**DOI:** 10.1016/j.ijscr.2019.10.025

**Published:** 2019-10-18

**Authors:** Hassan Nasser, Tommy Ivanics, Shravan Leonard-Murali, Jeffrey Genaw

**Affiliations:** Department of Surgery, Henry Ford Hospital, 2799 W Grand Blvd, Detroit, MI 48202, USA

**Keywords:** LAGB, laparoscopic adjustable gastric band, BE, band erosion, ERCP, endoscopic retrograde cholangiopancreatography, Laparoscopic adjustable gastric band, Gastric band complication, Intraluminal migration, Band erosion, Biliary obstruction, Case report

## Abstract

**Introduction:**

Laparoscopic adjustable gastric band is a bariatric operation which has lost popularity due to its high rate of reoperation and complications such as band erosion. Erosion may be partial or complete with intragastric migration of the band. Once in the stomach lumen, the band has the potential to migrate into the small bowel.

**Presentation of case:**

A 43-year-old male with history of morbid obesity and laparoscopic adjustable gastric band placement presented with abdominal pain secondary to biliary obstruction. Endoscopic retrograde cholangiopancreatography revealed eroded gastric band tubing into the lumen of the stomach and duodenum with resultant distortion of the ampulla. Upon surgical exploration, the band was found to have migrated into the jejunum and was removed through an enterotomy. The patient did well and was discharged home on postoperative day 8.

**Discussion:**

Once completely eroded into the gastric lumen, a gastric band can migrate into the small bowel with the distance traveled being limited by the length of the connecting tube. The stretched tubing can result in distortion of the ampulla leading to biliary obstruction. Band erosion should be managed with band removal which can be completed using endoscopic, laparoscopic, or open approach.

**Conclusion:**

Band migration should be suspected in patients with a history of gastric band placement presenting with bowel or biliary obstruction. Its management depends on the location of the band as well as the expertise of the surgical team.

## Highlights

• LAGB can rarely erode into the stomach and migrate into the small bowel.
• Migration of the LAGB can result in bowel and biliary obstruction.
• Band erosion should be managed by removal of the LAGB.

## 1 Introduction

Laparoscopic adjustable gastric band (LAGB) is a bariatric procedure that has lost popularity in the last decade due to a high rate of reoperation, complications, and weight regain [1]. At 10 years, up to 50% of patients with LAGB may require band removal with or without conversion to another bariatric procedure, usually a Roux-en-Y gastric bypass [1]. In the United States, LAGB represented only 3.4% of bariatric procedures performed in 2016 compared to 35.4% in 2011 [2]. Band erosion (BE), one of the more severe complications associated with LAGB, has a reported rate of 1.46% [3]. BE can be partial or complete with intragastric migration of the band [4]. When completely internalized into the gastric lumen, the band has the potential to migrate into the small bowel [[5], [6], [7], [8], [9]]. Biliary obstruction is a very rare complication that may result from intrajejunal band migration. We report a rare case of gastric band erosion and migration into the jejunum with resultant biliary obstruction from the stretched band tubing that was managed with laparotomy and band removal. This case reports has been reported in line with the surgical case report (SCARE) criteria [10].

## 2 Presentation of case

A 43-year-old male presented to the emergency department with epigastric abdominal pain and vomiting for 4 days. Seven years earlier he underwent a LAGB placement at another institution; his body mass index then was 46.8 kg/m^2^ versus 44.5 kg/m^2^ on this presentation. He was lost to follow-up a few years after band placement. His medical history was significant for diabetes mellitus controlled with metformin.

**Fig. 1:**
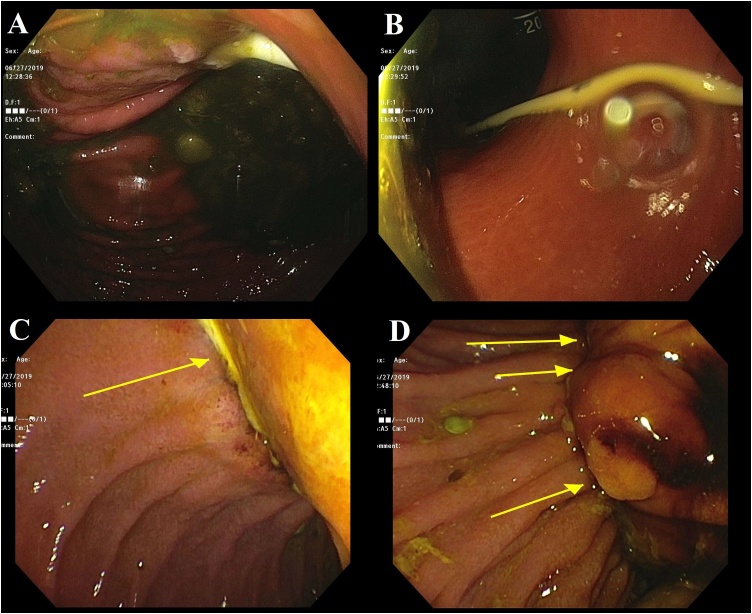
(A & B) Gastric band tubing noted in the stomach on endoscopy. (C) Gastric band tubing noted in the duodenum on endoscopy. (D) Tubing in the duodenum with distortion of the major papilla.

**Fig. 2:**
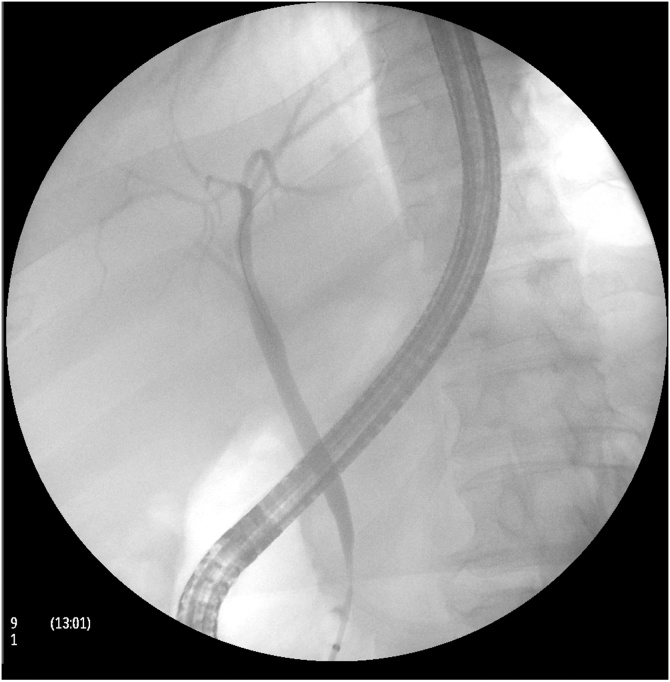
Endoscopic retrograde cholangiogram showing the 10 mm area of narrowing in the lower bile duct secondary to compression from the stretched band tubing.

Abdominal examination revealed tenderness in the epigastrium without signs of peritonitis. White blood cell count was 8800/μL, total bilirubin 7.2 mg/dL, direct bilirubin 4.4 mg/dL, and alkaline phosphatase 90 IU/L. Abdominal ultrasound revealed cholelithiasis in the gallbladder with a dilated common bile duct measuring 7.5 mm without evidence of acute cholecystitis. An endoscopic retrograde cholangiopancreatography (ERCP) was performed given the concern for choledocholithiasis. The ERCP revealed erosion of the gastric band tubing into the stomach and duodenum with secondary distortion of the major papilla (Fig. 1). The gastric band was not visualized in the lumen of the stomach. Cholangiogram revealed a 10 mm area of narrowing in the lower bile duct likely secondary to compression from the tubing (Fig. 2). During sphincterotomy, no stones were extracted from the biliary tree although there was a small amount of sludge. A plastic stent was placed in the common bile duct. Total and direct bilirubin decreased (3 and 1.3 mg/dL, respectively) after biliary drainage but did not normalize.

**Fig. 3:**
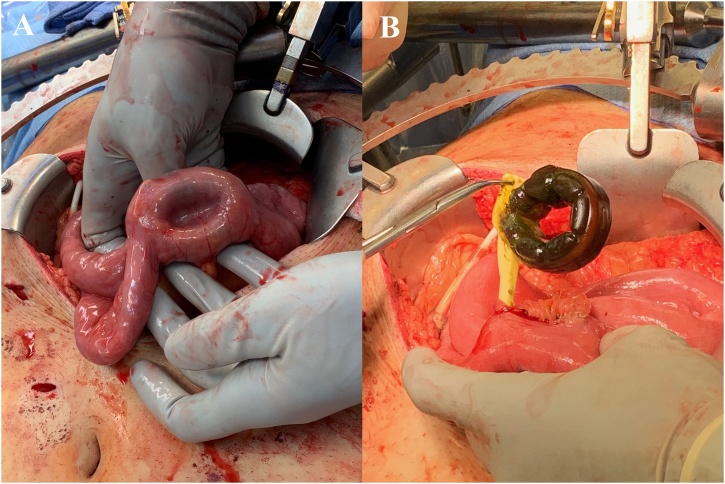
(A) Intraoperative image showing the gastric band in the lumen of the proximal jejunum. (B) Extraction of the gastric band through an enterotomy.

In view of the endoscopic findings, a decision was made with the patient to proceed with laparoscopic removal of the gastric band and cholecystectomy simultaneously. After the completion of an uneventful cholecystectomy, we proceeded to remove the gastric band. The band tubing was noted to be under tension and was cut. Adhesions from the stomach to the left hepatic lobe were taken down. An iatrogenic gastrotomy was made during the dissection. Despite extensive take down of the adhesions, we were unable to identify the gastric band on the stomach. An intraoperative esophagogastroduodenoscopy was performed to help identify the location of the band. The tubing, which had been noted on the prior ERCP, could not be visualized in the lumen and the band location could not be identified. On conversion to an open laparotomy, the small bowel was examined, and the band was identified in the proximal jejunum about 50 cm from the ligament of Treitz (Fig. 3A). The band was extracted through an enterotomy (Fig. 3B) that was repaired primarily. The gastrotomy was repaired in two layers and an adjacent 19F Jackson-Pratt drain was placed along with a nasogastric tube.

The patient had an uncomplicated postoperative course. His liver function tests normalized. On postoperative day 5, an upper gastrointestinal swallow study showed no leak. The nasogastric tube was then removed. The patient tolerated a diet and had return of bowel function. The patient was discharged home on postoperative day 8 with the drain in place. He was seen in clinic for drain removal on postoperative day 15 and was doing well.

## 3 Discussion

BE is a rare yet serious complication of LAGB. The most common symptoms of BE include abdominal pain, nausea, vomiting, weight regain, and port infection. Although the exact etiology is unclear, BE is presumed to be related to technical factors during initial placement and external pressure on the stomach applied by the band [3,11]. The incidence of BE decreases with surgeon experience [3]. The average duration from band placement to BE varies depending on length of follow-up; Quadri et al. reported an average time of 68.5 months [4].

Once eroded completely into the gastric lumen, the band can rarely migrate into the small bowel which may result in obstruction or perforation [8,9,12]. The distance traveled is limited by the length of the connecting tube. In our patient, the gastric band eroded into the stomach and migrated into the proximal jejunum while still tethered by the band tubing. The stretched tubing distorted the major papilla resulting in biliary obstruction, which was attributed mistakenly to choledocholithiasis prior to endoscopy. The patient’s bilirubin decreased after decompression and stenting of the biliary tree but did not return to normal until the band was removed. Shah et al. reported a case of intraluminal migration of an adjustable band after a vertical-banded gastroplasty with biliary obstruction although its mechanism was not clearly reported [8]. We failed to appreciate the migrated band on ERCP as it was past the duodenum and preoperative abdominal imaging was not obtained to help localize the band. When the tubing was cut during laparoscopy, the band migrated further into the small bowel dragging the band tubing along, which explained why we could not visualize the tubing in the lumen on intraoperative endoscopy.

When diagnosed, BE should be managed with band removal. The approach to intragastric migration depends on partial or complete erosion [4]. Endoscopic retrieval requires erosion of >50% of the band together with its lock [11]. However, successful removal of a migrated intrajejunal LAGB has been reported with the endoscopic, laparoscopic, and open approaches [6,7,9]. In our patient, we resorted to the open approach as band migration was not noted preoperatively and we could not locate the band laparoscopically.

## 4 Conclusion

Band migration is a rare complication that should be suspected in patients with a history of gastric banding presenting with bowel or biliary obstruction even after many years. Attempts should be made to localize the band and degree of erosion with preoperative imaging and endoscopy when there is any suspicion of band or tube erosion. The approach to management depends on the location of the band as well as the expertise of the surgical team.

## Sources of funding

No source to be stated.

## Ethical approval

The study is exempt from ethical approval in our institution.

## Consent

Written informed consent was obtained for publication of this case report and accompanying images. A copy of the written consent is available for review by the Editor-in-Chief of this journal on request

## Author contribution

Hassan Nasser, MD: Writing – original draft & review.

Tommy Ivanics, MD: Writing – review & editing.

Shravan Leonard-Murali, MD: Visualization.

Jeffrey Genaw, MD: Supervision.

## Registration of research studies

Not applicable.

## Guarantor

Jeffrey Genaw, MD.

## Provenance and peer review

Not commissioned, externally peer-reviewed.

## Declaration of Competing Interest

No conflicts of interest to be declared.
